# Global challenges of graduate level Ayurvedic education

**DOI:** 10.4103/0974-7788.64398

**Published:** 2010

**Authors:** Sanjeev Rastogi

**Affiliations:** *Department of Kaya Chikitsa, State Ayurvedic College and Hospital, Department of Holistic Medicine, BMCRC, Vatsala Hospital, Tulsi Das Marg, Lucknow, India E-mail: rastogisanjeev@rediffmail.com*

Sir,

The study by Patwardhan *et al*,[1] is one among many of the revealing studies carried out from time to time by researchers who are concerned about the plight of Ayurveda and are genuinely thinking about possible methods to make improvements in its system appraisal.

Why does a person trained institutionally in the Ayurvedic system of medicine not appear as confi dent about his future as his modern counterpart may seem to be?[1] The reasons could be many but they all essentially lead to a poor education system adopted for imparting training in Ayurveda in India. It is important to understand that to instruct students in Ayurveda so as to make them confi dent, we need to have a battery of good mentors. The administrative system of Ayurveda therefore needs to address the issue of fi nding and retaining people with essential skills that can augment the capacities of a student.

Without doubt, we need to understand that to improve our education system, we need to empower our teachers with skills and abilities to explore the subject so that they can comfortably engage in the betterment of Ayurveda.[2,3]
